# Caregiver Burden Domains and Their Relationship with Anxiety and Depression in the First Six Months of Cancer Diagnosis

**DOI:** 10.3390/ijerph17114101

**Published:** 2020-06-09

**Authors:** Francisco García-Torres, Marcin J. Jabłoński, Ángel Gómez Solís, María José Jaén-Moreno, Mario Gálvez-Lara, Juan A. Moriana, María José Moreno-Díaz, Enrique Aranda

**Affiliations:** 1 Department of Psychology, University of Córdoba, 14071 Córdoba, Spain; z12gatof@uco.es (F.G.-T); jamoriana@uco.es (J.A.M.); 2 IMIBIC Health Research Institute, Reina Sofía University Hospital of Córdoba, 14004 Córdoba, Spain; 3 Institute of Psychology, Faculty of Philosophy, Jesuit University Ignatianum in Krakow, 31501 Kraków, Poland; marcinjablonski@interia.pl; 4 Reina Sofía University Hospital of Córdoba, 14004 Córdoba, Spain; angelgomezsolis@gmail.com; 5 IMIBIC Health Research Institute, Department of Social Health Sciences, Radiology and Physical Medicine, University of Córdoba, 14005 Córdoba, Spain; mjjaen@gmail.com; 6 Department of Social Health Sciences, Radiology and Physical Medicine, University of Córdoba, 14004 Córdoba, Spain; arracada@gmail.com; 7 Medical Oncology Department, Reina Sofía University Hospital, 14004 Córdoba, Spain; earandaa@seom.org

**Keywords:** cancer, oncology, caregivers, burden, anxiety, depression

## Abstract

Cancer caregiving is associated with burden and a poor psychological state. However, there is no previous information about the predictive utility of specific burden domains on anxiety and depression in the first six months after a partner’s cancer diagnosis. In a longitudinal study, 67 caregivers completed the Zarit Burden Interview (ZBI) and Hospital Anxiety and Depression Scale (HADS) at T1 (45–60 days after diagnosis) and T2 (180–200 days after diagnosis). Most of the caregivers were female (65.7%, mean age = 51.63, *SD* = 13.25), while patients were mostly male (56.7%). The TRIPOD checklist was applied. ZBI scores were moderate and HADS anxiety reached significant values. There were no differences in ZBI and HADS between T1 and T2. The relationship between burden, anxiety, and depression were more consistent at T2, while emotional burden at T1 were related and predicted anxiety and depression at T2. Some burden domains were related and predicted anxiety in caregivers in the first six months after partner cancer diagnosis. This information could be useful to prevent the onset of these symptoms in the first six months after diagnosis.

## 1. Introduction

Cancer is still a major health problem worldwide. The number of new cancer diagnoses increased from 14 million in 2012 to 18.1 million in 2018. It is expected that in 2019 there will be around 277,234 new cancer diagnoses in Spain, being the second leading cause of death (26.7%) behind cardiovascular disease [1]. This growing number of patients and cancer survivors brings new challenges not only to patients but to caregivers of patients during and after diagnosis of the disease. Informal caregivers can be defined as individuals (e.g., spouse, parent, friend, neighbor) who provide care to cancer patients, which implies the need to devote a large amount of time and effort for extended periods of time in tasks that are very demanding in different areas (e.g., social, emotional, financial). It is also necessary to mention that most caregivers do not receive training that would provide them with adequate strategies to cope with this new situation, which often leads to the emergence of intense feelings of discomfort or even burden [2,3]. Caregiver burden can be described as the perception of the degree to which the physical health and psychological well-being, social life, and financial status are affected by a close relative with cancer [4].

In cancer patients, there are certain particularities that differ from other diseases and that differentially influence caregivers. For example, it is possible that the disease causes a rapid deterioration in a short time in the patient, which can give rise to intense distress in close family and friends [5]. In addition, cancer patients can experience wide variability in the symptoms associated with the disease, which implies greater dedication of caregivers in terms of time and effort compared to other types of diseases [6]. This situation can lead to the presence of significant feelings of burden in informal caregivers of cancer patients, with different needs for support, particularly in the first six months when the majority of patients undergo anti-cancer treatments [7,8]. In general, it is well established that burden in caregivers is frequently associated with high psychological distress early after diagnosis [9,10,11,12,13]. Previous research has shown an association between burden and anxiety/depression in cancer caregivers [14,15,16], indicating that high burden at diagnosis is related to anxiety and depression [17]. Moreover, previous data shows the predictive capacity of burden on depression in caregivers of hospitalized advanced cancer patients [18], but most of these results were obtained in cross-sectional studies, limiting the generalization of the results obtained and not specifically focused in the first months after cancer diagnosis. In a longitudinal study carried out with partners of men diagnosed with prostate cancer, the results showed that in the first six months of the treatment, anxiety and depression did not change over time, but a significant subgroup of participants (23–25%) experienced anxiety, and higher caregiver burden was strongly associated with poor psychological state at all assessment points [19]. In caregivers of patients with lung cancer, the results indicated that psychological distress was lower at six months compared with baseline, while caregiver burden remained stable and even increased over time, showing associations with psychological distress at six months [20]. Despite these promising results, they are limited to a specific cancer diagnosis, so it could be useful to include a wide variety of cancer types to achieve a better understanding of caregiver burden and psychological distress relations within the first six months of cancer. On the other hand, there is little information about the relations between specific burden domains and anxiety and depression in cancer caregivers. Previous research has shown that in caregivers of lung cancer, the burden dimension most affected was “impact on daily schedule” [8], but in general there is little information about the specific burden domains that are most affected in caregivers within the first six months of care. Considering that caregiving can be burdensome in a wide variety of situations in the daily lives of caregivers [2,3,21], this could be necessary to obtain a better understanding about the ways in which burden negatively influences caregivers within this period.

Therefore, this longitudinal study has the following objectives. First, caregiver burden, anxiety, and depression were assessed at two time points, T1 (45–60) days after diagnosis) and T2 (180‒200 days after diagnosis), to examine the differences and relations between these variables at the two time points separately. Second, the predictive utility of burden domains at diagnosis (T1) on anxiety and depression scores observed six months later (T2) were tested.

## 2. Materials and Methods 

### 2.1. Participants

**Figure 1:**
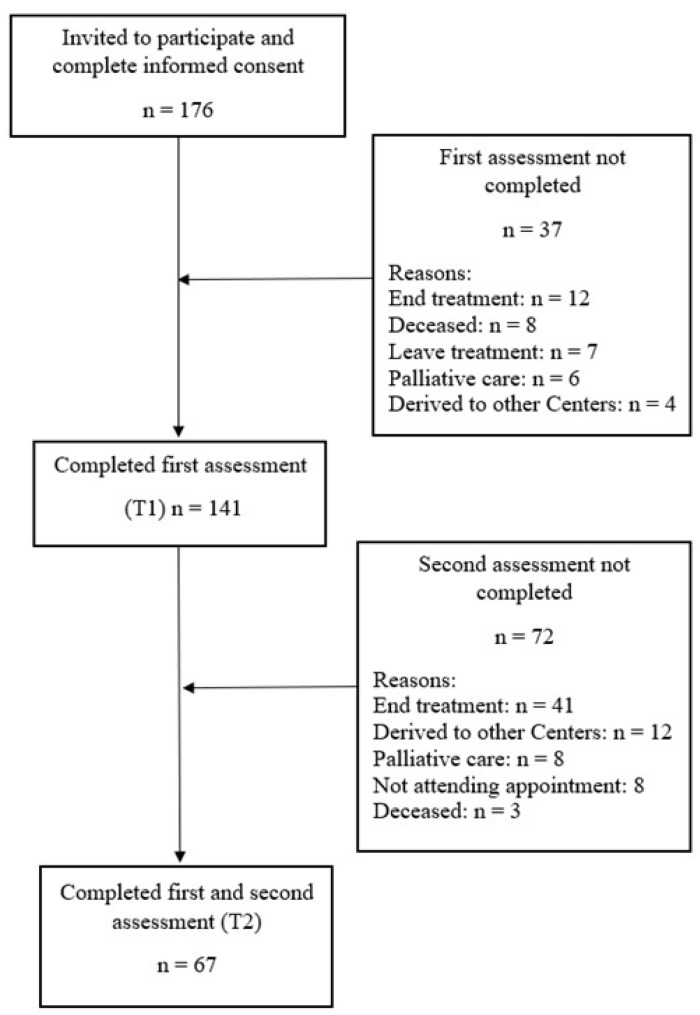
Recruitment flowchart of the participants in the study.

A total of 176 caregivers and 176 patients were invited to participate in the study. The average age of caregivers was 51.63 years (*SD* = 13.25) and of patients was 58.60 years (*SD* = 15.28). The inclusion criteria for caregivers were as follows: men and women (at least 21 years old) currently in a relationship with a person diagnosed with cancer 30–45 days prior; mentally capable of answering the questionnaires with no history of dementia or intellectual disability; living in the same household with the patient; and not a professional caregiver. The inclusion criteria for patients were having a cancer diagnosis (all types), being eligible for treatment, and being at least 21 years old. Caregivers and patients signed their informed consent prior to data collection. The selection and data collection were carried out between March 2017 and November 2018 at Reina Sofía Hospital in Córdoba, Spain, in the day center unit for ambulatory cancer patients. Of the 176 caregivers and patients who were invited to participate in the study, 141 completed the first assessment, resulting in a response rate of 80%. The second assessment was completed by 67 caregivers (47% response rate). This response rate is similar to those observed in studies with caregivers [13,20], and the sample size is similar to previous published research [7,18]. Detailed recruitment information is shown in Figure 1.

### 2.2. Variables and Instruments

First, patients and caregivers completed a questionnaire on clinical and sociodemographic data, including sex, age, education, employment status, and relation with patient. Patients also completed information about their cancer type and treatment. Clinical characteristics (cancer stage) were obtained from clinical records. Second, caregivers completed the Zarit Burden Interview (ZBI) [4]. This questionnaire is widely used to assess burden in cancer settings. It is composed of 22 items with answers ranging from 1 (never) to 5 (nearly always) in which higher scores indicate a higher burden. There is no consensus on the names and number of domains for the 22-item ZBI. It reflects the diversity of approaches in studies/factor analysis/global analysis, the diversity of samples (type of caregivers) and sample sizes, the diversity of disease characteristics of patients in interaction with caregivers, and the cultural differences in which the studies are undertaken. In accordance with the recommendations of the MAPI Research Trust, which owns the ZBI license and the proposal of Whitlatch, Zarit, and Von Eye [22], the following burden dimensions were obtained: burden in the relationship, emotional burden, social and family life, finances, loss of control over one’s life, personal strain, and role strain. This questionnaire has shown excellent internal consistency in a Spanish sample (*α* = 0.91) [23]. Caregivers also completed the Hospital Anxiety and Depression Scale (HADS) [24]. This instrument assesses anxiety and depression through two subscales with seven items each, scored from 0–3. The maximum score is 21 for both subscales. This scale establishes a score of 8 as a cut-off point for anxiety (HADSA) and depression (HADSD). The internal consistency of this instrument was shown to be good in a Spanish population for HADSA (*α* = 0.86) and HADSD (*α* = 0.86) [25].

### 2.3. Procedure

All possible participants who met the inclusion/exclusion criteria were invited to participate in the study. Caregivers and patients were consecutively recruited by trained nurses within 45 days after the partner’s diagnosis of cancer. Once the possible participants (caregivers and patients) agreed to collaborate, an appointment with a member of the research team was scheduled to collect data 45–60 days after diagnosis (T1). The second observation (T2) was performed 180–200 days after diagnosis. Data collection was supervised by the nurse staff and was established to coincide with the follow-up appointments during the first six months of cancer treatment. The nurses gave the set of questionnaires to the participants to complete in the waiting room during the appointments. The study was approved by the ethics committee of the Portal of Ethics of Biomedical Research of Andalusia (ref. no. 3262). All participants completed an informed consent form prior to participating in the study, which assured the confidentiality of the results and established that participants could leave the study at any time without reason and with no negative consequences. TRIPOD checklist was completed (Transparent Reporting of Multivariate Prediction Model for Individual Prognosis or Diagnosis Statement (See Supplementary File 1)).

### 2.4. Statistical Analysis

Descriptive statistics were obtained from the sociodemographic and clinical data of the whole sample. Differences between T1 and T2 were assessed using paired-samples *t*-tests on ZBI and HADS scores. Pearson correlations were used to establish the relations between ZBI domains and HADS anxiety and depression scores at T1 and T2 and to test the relations between ZBI domains at T1 on HADS scores at T2. Finally, multiple regression analysis were performed with HADS anxiety and depression scores (T2) as outcomes and the burden dimension (T1) as predictor, controlling for cancer type, treatment, cancer stage, employment status and type of relationship in the first block using forced entry and the burden dimensions in the second block using the stepwise method. Sample size calculation for regression models were performed using G*Power [26]. Previous data have shown a squared multiple correlation of 0.41 and 0.47, using depression and anxiety respectively as DV and ZBI as IV [18]. Estimating a *F* test priori analysis, linear multiple regression: fixed model, *R*^2^ deviation from zero, with a power of 0.80 and α = 0.05, the results showed that a minimum sample size between 26–31 patients is required. Previous similar research have shown an attrition rate of 31% [20], a comparable sample size in our study would be around 34–41 participants. The results were accepted as significant with *p* ≤ 0.05. 

## 3. Results

Information about the caregivers and patient characteristics is shown in Table 1. Most of the caregivers were women (65.7%), and the most common relationship with the patient was partner (58.2%). In patients, the most common cancer type was gastrointestinal (49.2%), and surgery plus chemotherapy (42.4%) was the most frequent treatment. 

In terms of the burden experienced by the caregivers, the overall burden at T1 and T2 can be categorized as moderate. In the anxiety and depression HADS scores, the results obtained at T1 and T2 were similar, and only anxiety can be considered as clinically relevant. There were no significant differences in ZBI and HADS scores comparing T1 with T2 (see Table 2).

Pearson correlations between burden domains and HADS anxiety and depression scores at T1 and T2 independently are shown in Table 3. The relations were more consistent at T2 where almost all burden domains were related to anxiety and depression (see Table 3).

Assessing the relations between burden domains at T1 with HADS scores at T2, only the dimension of emotional burden at T1 was related with HADSA (*r*(67) = 0.24, *p* = 0.04) and HADSD (*r*(67) = 0.29, *p* = 0.01) scores at T2. Finally, multiple regression analysis showed the predictive capacity of the ZBI domain of emotional burden (T1) on anxiety and depression scores assessed at T2 (see Table 4 and Table 5).

## 4. Discussion

Informal caregivers might experience burden as a consequence of cancer diagnosis in a close relative [9,10,11,13]. There is little information, however, about the relations and the predictive utility of the different domains of burden for anxiety and depression in the first six months after diagnosis using longitudinal studies with different cancer types. Previous research has shown moderate levels of burden experienced for caregivers within this period [8,20]. In our study, a similar pattern is observed with moderate levels of burden, showing similar scores at the two assessment points. It is possible that the average age of the sample (around 50 years old) influenced these results, as it is observed that younger age is related to higher caregiving burden [27]. In anxiety and depression, only anxiety scores reached clinically significant values but with no statistical differences between the two assessment points. In our study, most of the caregivers were female, and these results are similar to those observed previously by previous research on female caregivers, so it is possible that this pattern of emotional response is related to the gender of the sample [22].

Previous data have shown that burden is related to anxiety and depression in caregivers [14,15,16]. The results obtained suggest a similar response, but analyzing the two assessment points independently, some differences in the relations between burden domains and anxiety/depression appears. Shortly after cancer diagnosis, the relations between burden and anxiety/depression were observed in the dimensions of loss of control and personal strain only, but six months later, almost all domains of burden (excluding role strain) were strongly related with anxiety and depression. These results suggest that during the first months of cancer care, the assumption of the new caregiving role negatively influences a wide variety of aspects in daily life that can be experienced as burdensome, but there are differences over time probably related to the duration of caregiving. During the first months of care, critical changes happen in patients’ and caregivers’ lives that affect a wide range of life situations [14,19]. Furthermore, emotional burden evaluated within the first 45 days after diagnosis is related and predicts anxiety and depressive symptoms over 180–200 days later, so it appears that the emotional dimension of burden is a key concept in the development of psychological distress in caregivers within this period. These results are different from those observed in caregivers of hospitalized cancer patients [18], probably due to the impact of the assumption of caregiving duties in ambulatory patients in the first months of cancer care for caregivers in terms of the high amount of new task to face without previous training, uncertainty about the future and the high presence of women acting as informal caregivers [28,29]. These results on the predictive utility of several domains of burden for anxiety in caregivers within the first six months after treatment are novel and could help to achieve a better understanding of how caregiving influences the psychological state of caregivers and the need to support caregivers in specific caregiving domains after an acute period of diagnosis and treatment, with the aim to develop specific interventions that prevent the development of emotional distress such as for example CHESS program (Comprehensive Health Enhancement Support System) [30]. Moreover, clinicians and nursing staff must pay attention to these relations to prevent the apparition of emotional distress in caregivers within the first six months of cancer care when distress appears more frequently, designing effective interventions not only to improve caregivers’ psychological well-being but also to allow cancer patients to adjust to their illness.

Finally, is it necessary to highlight some limitations of the study. Firstly, the sample size was limited, so it would be necessary to expand the sample to generalize the results obtained, but our results are similar to those observed in other studies with similar sample sizes [7,18]. In this line, the study was conducted in Spain, so this may limit the generalization of the results. The variety of cancer types and treatments can also be considered, and the different prognosis of the patients must be controlled in future research. Furthermore, the social/financial status of the sample may influence the results. Future research must consider the relevance of considering a more specific analysis of burden and emotional reactions in caregivers of cancer patients. 

## 5. Conclusions 

This longitudinal study identified specific burden domains, emotional burden, that are related with psychological distress in caregivers in the first months of cancer care. This information may be useful in order to prevent the apparition of anxiety and depression symptoms in cancer caregivers and to develop specific intervention in the early stage of cancer treatment for cancer caregivers. 

## Figures and Tables

**Table 1:** Clinical and sociodemographic data of the sample.

	Caregivers	Patients
**Sex**		
Female	44 (65.7%)	38(56.7%)
Male	23 (34.3%)	29 (43.3%)
**Age M (*SD*)**	51.63 (13.25)	58.60 (15.28)
**Education**		
Basic	35.8%	41.8%
Vocational	26.9%	23.9%
Secondary	14.9%	13.4%
Collegue	22.4%	20.9%
**Employment status**		
Full time job	32.8%	23.4%
Own business	16.4%	9.4%
Seasonal work	20.9%	17.2%
Unemployment	19.4%	17.2%
Rent	1.5%	4.7%
Retirement	9%	28.1%
**Relation with patient**		
Partner	58.2%	
Father/Mother	3%	
Son/Daughter	28.4%	
Brother/Sister	7.5%	
Friend	3%	
**Cancer type**		
Head and neck		10.8%
Lung		6.2%
Breast		21.5%
Gastrointestinal		49.2%
Uterine/Ovarian		3.1%
Genitourinary		6.2%
Conective tissue/Skin		3.1%
**Cancer stage**		
I		17.9%
II		23.9%
II		17.9%
IV		40.3%
**Treatment type**		
Surgery		12.1%
Chemotherapy		18.2%
Radiotherapy		1.5%
Hormonal		1.5%
Surgery + Chemotherapy		42.4%
Surgery + Radiotherapy		1.5%
Surgery + Chemotherapy + Radiotherapy		22.7%

**Table 2:** ZBI and HADS scores at T1 and T2 and *t* test results.

ZBI Domains (Max Poss. Score)	T1 *M*(*SD*)	T2 *M*(*SD*)	*t*	*p*
Total burden (88)	42.66 (12.77)	41.16 (12.06)	1.41	0.16
Burden in the relationship (24)	12.72 (4.09)	12.15 (3.08)	1.75	0.08
Emotional burden (28)	11.94 (3.97)	11.73 (3.52)	0.54	0.58
Social and family life burden (16)	7.67 (3.31)	7.37 (2.93)	1.19	0.23
Financial burden (4)	1.84 (1.06)	1.63 (.75)	1.80	0.07
Loss of control over one’s life (16)	8.61 (3.00)	8.28 (2.71)	1.12	0.26
Personal strain (48)	22.67 (6.87)	21.84 (6.41)	1.45	0.15
Role strain (24)	11.64 (4.71)	11.06 (4.17)	1.56	0.12
**HADS scores**	**T1 *M*(*SD*)**	**T2 *M*(*SD*)**	***t***	***p***
HADSA (21)	8.24 (4.06)	8.45 (3.23)	−0.53	0.59
HADSD (21)	6.40 (3.91)	7.00 (3.28)	−1.47	0.14

HADSA = Hospital Anxiety and Depression Scale Anxiety; HADSD = Hospital Anxiety and Depression Scale-Depression; ZBI = Zarit Burden Interview. Max Possible Score: Maximum possible score.

**Table 3:** Relationships between burden domains and HADS scores in T1 and T2.

ZBI Domains (T1/T2)	T1		T2	
	HADSA	HADSD	HADSA	HADSD
Total Burden	0.18	0.22	0.33 **	0.33 **
Burden in the relationship	0.12	0.12	0.28 *	0.24 *
Emotional burden	0.12	0.20	0.31 *	0.35 **
Social and family life burden	0.17	0.17	0.21	0.24 *
Financial burden	−0.02	−0.10	0.28 *	0.26 *
Loss control over one´s life	0.27 *	0.33 **	0.38 **	0.37 **
Personal Strain	0.14	0.24 *	0.36 **	0.34 **
Role strain	0.16	0.13	0.18	0.21

T1 (45–60: days after diagnosis/T2: 180–200 days after diagnosis). * *p* < 0.05, ** *p* < 0.001.

**Table 4:** Regression analysis results for emotional burden (ZBI) on anxiety (HADSA).

Anxiety	*b*	SE *b*	*β*	*t*
Model 1				
Constant	9.78	1.98		4.92 ***
Cancer type	−0.04	0.29	−0.02	−0.15
Treatment	−0.18	0.18	−0.13	−1.00
Cancer stage	0.27	0.34	0.10	0.80
Type of relationship	−0.26	0.33	−0.10	−0.78
Employment status	−0.34	0.24	−0.18	−1.40
Model 2				
constant	7.02	2.19		3.19 **
Cancer type	−0.09	0.28	−0.04	−0.33
Treatment	−1.34	0.18	−0.09	−0.73
Cancer stage	0.25	0.33	0.10	0.77
Type of relationship	−0.38	0.32	−0.15	−1.18
Employment status	−2.87	0.23	−0.15	−1.20
Emotional burden	0.23	0.09	0.31	2.50 *

*R*^2^ = 0.06 for Step 1; ∆*R*^2^ = 0.093 for Step 2 (*p* < 0.05). * *p* < 0.05, ** *p* < 0.01, *** *p* < 0.001.

**Table 5:** Regression analysis results for emotional burden (ZBI) on depression (HADSD).

Depression	*b*	SE *b*	*β*	*t*
Model 1				
Constant	9.22	2.01		4.57 ***
Cancer type	0.04	0.27	0.01	0.14
Treatment	−0.24	0.19	−0.16	−1.27
Cancer stage	−0.03	0.35	−0.01	−0.10
Type of relationship	−0.38	0.34	−0.14	−1.10
Employment status	−0.26	0.25	−0.13	−1.04
Model 2				
constant	6.00	2.19		2.74 **
Cancer type	−0.01	0.28	−0.007	−0.95
Treatment	−0.18	0.18	−0.12	−0.99
Cancer stage	−0.59	0.33	−0.02	−0.18
Type of relationship	−0.52	0.32	−0.20	−1.59
Employment status	−0.19	0.23	−0.09	−0.79
Emotional burden	0.27	0.09	0.36	2.92 **

*R*^2^ = 0.05 for Step 1; ∆*R*^2^ = 0.12 for Step 2 (*p* < 0.01). ** *p* < 0.01, *** *p* < 0.001.

## Supplementary Materials

The following are available online at https://www.mdpi.com/1660-4601/17/11/4101/s1, Supplementary File 1. TRIPOD Checklist: Prediction Model Development.
